# The Prevalence of Depression among Diabetic Patients in Ethiopia: A Systematic Review and Meta-Analysis, 2018

**DOI:** 10.1155/2018/6135460

**Published:** 2018-05-23

**Authors:** Henok Mulugeta Teshome, Getenet Dessie Ayalew, Fasil Wagnew Shiferaw, Cheru Tesema Leshargie, Dube Jara Boneya

**Affiliations:** ^1^Department of Nursing, College of Health Sciences, Debre Markos University, Debre Markos, Ethiopia; ^2^Department of Public Health, College of Health Sciences, Debre Markos University, Debre Markos, Ethiopia

## Abstract

**Background:**

Diabetes mellitus is a chronic metabolic disorder characterized by hyperglycemia. Depression is one of the major important public health problems that is often comorbid with diabetes. Despite the huge effect of comorbid depression and diabetes, the overall pooled prevalence of depression among diabetic patients in the country level remains unknown. Therefore, the objective of this systematic review and meta-analysis is to estimate the pooled prevalence of depression among patients with diabetes mellitus in Ethiopia.

**Method:**

Data extraction was designed in accordance with the Preferred Reporting Items for Systematic Reviews and Meta-Analyses (PRISMA) guidelines. Studies were accessed through electronic web-based search from PubMed, Cochrane Library, Google Scholar, Embase, and PsycINFO. All statistical analyses were done using STATA version 11 software using random effects model. The pooled prevalence was presented in forest plots.

**Results:**

A total of 9 studies with 2944 participants were included in this meta-analysis and the overall pooled estimated prevalence of depression among diabetic patients in Ethiopia was 39.73% (95% CI (28.02%, 51.45%)). According to subgroup analysis the estimated prevalence of depression in Addis Ababa was 52.9% (95% CI: 36.93%, 68.88%) and in Oromia region was 45.49% (95% CI: 41.94, 49.03%).

**Conclusion:**

The analysis revealed that the overall prevalence of comorbid depression among diabetic patients in Ethiopia was high. Therefore, Ministry of Health should design multisectorial approach and context specific interventions that address this comorbid depression in this specific group as well as general population.

## 1. Background

Diabetes mellitus is a chronic metabolic disorder characterized by hyperglycemia with disturbances of carbohydrate, protein, and fat metabolism [1]. The global prevalence of diabetes has been increasing rapidly. In 2014, the overall global prevalence of diabetes among adult is 8.5%. In addition, according to world health organization report globally around 170 million people are affected by diabetes and this figure is more likely to be double by 2030. In 2015, an estimated 1.6 million deaths were directly caused by diabetes [2–4].

Today, the burden of diabetes remains one of the major public health important problems in Africa which have been resulting in high mortality and morbidity. Diabetes is a common chronic medical condition in sub-Saharan Africa countries including Ethiopian population. However, the overall incidence and prevalence of diabetes in Ethiopia are unknown due to very limited studies in the country [5, 6].

Depression, a common mental disorder characterized by persistent unhappiness and lack of interest in daily activities, is one of the major important public health problems that is often comorbid with other chronic diseases like diabetes and can worsen the effect of the disease outcomes. Depression alone and/or as comorbidity with diabetes is a common condition in the community [7–9].

The cause of depression in patients with diabetes is may be associated with the burden of complications, financial stress, poor overall health status, knowledge of diabetes, and poor glycemic control. The presence of depression in diabetic patients worsens the prognosis of diabetes, increases the noncompliance to the medical treatment, decreases the quality of life, and increases mortality. Moreover, the burden of depression in patients with diabetes is linked with increased diabetes complications, high mortality rates, and poor quality of life [10–12].

Different literature review and meta-analysis conducted worldwide showed that depression is a common comorbid condition among patients with diabetes [13–15]. A systematic literature review done to estimate the prevalence of comorbid depression in adults with type 1 diabetes showed that the prevalence of depression was 12.0% for people with diabetes [16]. Similarly, a systematic review and meta-analysis conducted in University Hospitals of Leicester revealed that the overall prevalence of depression was 17.6% in patients with diabetes [17].

The few studies conducted in Ethiopia have shown that the coexistence of diabetes and depression is highly prevalent in which depression was found to remain as an important comorbid condition with diabetes. Additionally, depression is highly prevalent among diabetes patients and associated with a number of debilitating conditions such as presence of diabetic complications, comorbidity, and the disease duration. Even this, few studies conducted on the prevalence of depression among diabetic patients in Ethiopia presented controversial and inconclusive findings [18–20].

Despite the huge effect of comorbid depression and diabetes and its importance as a public health problem in Ethiopia, the overall prevalence of depression among diabetes patient in the country level remains unknown. In addition, measuring of depression alone or as a comorbidity in patients with diabetes promotes the overall health status, avoids negative effect of depression, and even may prevent diabetes-related complications. Therefore, the objective of this systematic review and meta-analysis is to estimate the pooled prevalence of depression among patients with diabetes mellitus in Ethiopia.

## 2. Methods

### 2.1. Study Design and Search Strategy

A systemic review and meta-analysis was conducted using published and unpublished research on the prevalence of depression among patients with diabetes mellitus in Ethiopia. Our literature search strategy, selection of publications, data extraction, and the reporting of results for the review were designed in accordance with the Preferred Reporting Items for Systematic Reviews and Meta-Analyses (PRISMA) guidelines [21]. Both published and gray literatures on depression among diabetes patients were reviewed using the following major databases; PubMed, Cochrane Library, Google Scholar, CINAHL, Embase, and PsycINFO. The search for published studies was restricted to articles published in English language, by the age of the study participants (adult with diabetes) whose age is greater than 18 and the study country (study conducted only in Ethiopia) but it was not restricted by time. All published and unpublished articles up to November 2017 were included in the systematic review. Systematic literature searches were conducted from June to November 2017. During comprehensive literature search, the following search terms were used: “prevalence of depression among diabetes in Ethiopia”, “depression OR diabetes in Ethiopia”, “depression AND diabetes in Ethiopia”. Additionally, we observed the reference lists of published studies to identify additional articles.

### 2.2. Study Selection and Eligibility Criteria

In this systematic review and meta-analysis, we included all studies that were conducted on the prevalence of depression among diabetic patients regardless of the types of diabetic mellitus. The participants were adult with diabetes whose age is greater than 18 years, regardless of their sex and other characteristics. We included all study types that were published in the form of journal articles, master's thesis, and dissertations in English. Furthermore, literatures which failed to report prevalence of depression and those studies conducted on children were excluded. Since the number of articles in Ethiopia is relatively limited, no restriction was made for date of publication. But, studies that were not conducted in Ethiopia were also excluded.

### 2.3. Quality Assessment and Critical Appraisal

In this systematic review and meta-analysis the qualities of each article were assessed by using a critical appraisal tool for use in systematic reviews for prevalence study [22]. The methodological quality and eligibility of the identified articles were also assessed by three reviewers (H.M., G.D., and F.W.) and disagreements among reviewers were fixed accordingly with discussion. A modified version of the Newcastle-Ottawa Scale for cross-sectional study adapted from Modesti et al. [23] was also used to evaluate the quality of studies.

### 2.4. Data Extraction

After the search was conducted, data were extracted using prepiloted data extraction form which were developed by the authors. It included name of author, year of publication, region in the country, study design, response rate sample size, number of people with outcome, and overall prevalence. H.M. and F.W. conducted the primary data extraction and then G.D., D.J., and C.T. examined the extracted data independently. Any disagreement and inconsistencies were resolved by discussion and consensus.

### 2.5. Data Analysis and Synthesis

The extracted data were entered into computer through command window of STATA v.11 and the analysis was performed using STATA v.11. A random effects model was used to estimate the overall pooled prevalence. An important statistical issue in meta-analysis is handling of heterogeneity among studies. DerSimonian and Laird method, which assumes heterogeneity across studies, is the most common method for using random effects model in meta-analysis [24, 25]. A random effects meta-analysis is also recommended for use when heterogeneity between studies exists [26]. Data manipulation and all statistical analyses were done using STATA software, version 11.

The heterogeneity of studies was checked using *I*^2^ test statistics. *I*^2^ statistics is used to quantify the percentage of total variation in study estimate due to heterogeneity [27]. *I*^2^ statistics ranges from 0 to 100 percent. A value of 0% indicates no observed heterogeneity while 100% indicates significant heterogeneity. A *p* value less than 0.05 was used to declare heterogeneity [28, 29]. In this meta-analysis, *I*^2^ values were found to be high (>75%). Since this value is definite indicative of significant high heterogeneity, we conducted the analysis with a random effects model with 95% CIs as opposed to the fixed effect model to adjust for the observed variability. Moreover, presence of heterogeneity was also assessed by subgroup analysis and metaregression.

Visual assessment of publication bias was conducted using funnel plot. Asymmetry of the funnel plot is an indicator of publication bias [15]. Egger's and Begg's tests were also conducted to check potential publication bias. A *p* value less than 0.05 was used to declare statistical significance of publication bias [30]. Additionally, sensitivity analysis was also done to assess whether the pooled prevalence estimates were influenced by individual studies.

## 3. Results

### 3.1. Selection and Identification of Studies

**Figure 1:**
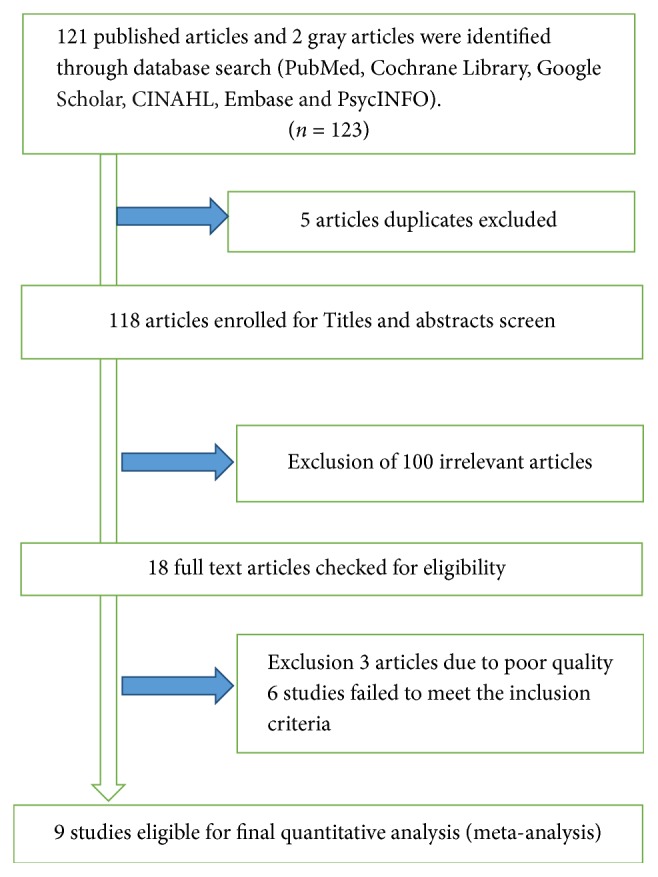
Flowchart of study selection for meta-analysis of prevalence of depression among diabetic patients in Ethiopia, 2018.

A total of 121 studies were identified from the literature search. We added two gray literatures that were not found in the search. Of these studies, 5 articles duplicate records were identified and removed. Reviewing of titles and abstracts resulted in exclusion of 100 irrelevant articles. After assessing the full texts of the remaining articles, additional 3 articles were excluded due to poor quality. Moreover, based on the inclusion and exclusion criteria for entry into the study a total of 6 studies were excluded as they did not meet the inclusion criteria. Then, a total of nine unique studies were eligible and enrolled for final analysis (Figure 1).

### 3.2. Characteristics of Included Studies

A total of 9 studies with 2944 participants included in this meta-analysis are summarized in Table 1. The studies were conducted from 2013 to 2017 in different regions of the country. Among 9 studies two of them [20, 31] were conducted in Addis Ababa, three study [18, 32, 33] were in Amhara region, two studies [12, 19] were in Oromia region, and the other two studies [34, 35] were in other regions of the country. All studies were cross-sectional study conducted among adult diabetic patients in Ethiopia. The study with minimum and maximum sample size was conducted in Amhara and Oromia region, respectively [12, 32]. In addition, out of all studies enrolled in this meta-analysis six studies [12, 18, 31, 33–35] were conducted among both type 1 and type 2 diabetic patients while the remaining four studies [1, 32] were conducted only among type 2 diabetic patients (Table 1).

### 3.3. Prevalence of Depression among Diabetes Mellitus Patients (Meta-Analysis)

**Figure 2:**
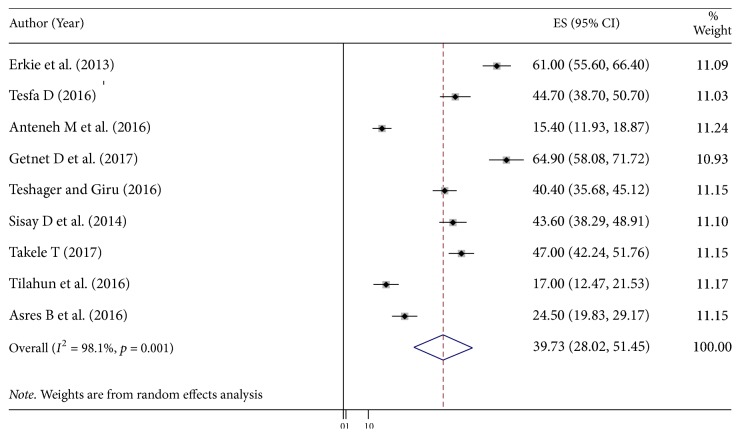
Forest plot showing the pooled prevalence of depression among diabetic patients in Ethiopia, 2018.

The pooled prevalence using the fixed effect model showed significant heterogeneity between the studies. Hence, we performed the analyses using random effects model. Using random effects model, the estimated pooled prevalence of depression among diabetic patients reported by the 9 studies was 39.73% (95% CI (28.02%, 51.45%)) with significant heterogeneity between studies (*I*^2^ = 98.1%, *p* = 0.001). The pooled prevalence of depression presented using forest plot (Figure 2).

**Figure 3:**
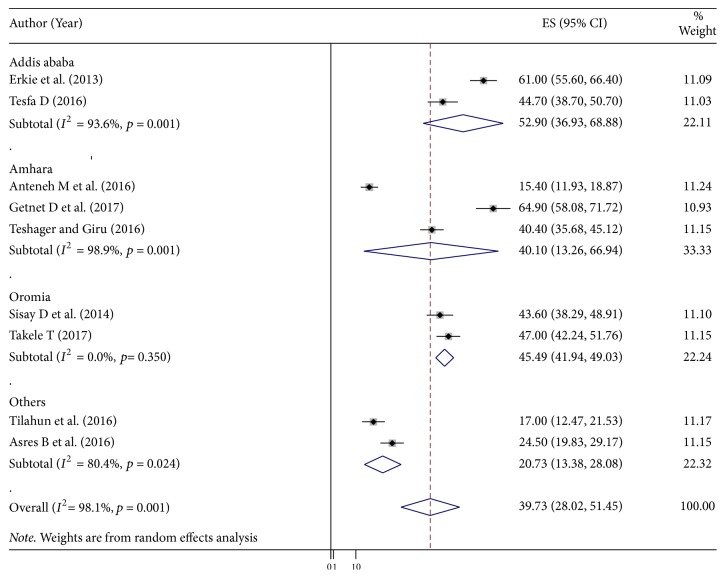
Subgroup analysis by regions on the prevalence of depression among diabetic patients in Ethiopia, 2018.

Subgroup analysis by study area was conducted to assess the potential heterogeneity between studies. Of the 9 studies, the highest estimated depression prevalence found in studies conducted in Addis Ababa (52.9% (95% CI: 36.93% to 68.88%), *I*^2^ = 93.6%), followed by studies conducted in Oromia region, was 45.49% (95% CI: 41.94, 49.03%), *I*^2^ = 0.0% (Figure 3).

### 3.4. Investigation of Heterogeneity

Heterogeneity in systematic reviews and meta-analysis results of studies is inevitable due to different in study quality, methodology, sample size, and inclusion criteria for participants [36, 37]. In this meta-analysis the value of *I*^2^ is definite indicative of significant high heterogeneity, so we conducted the analysis with a random effects model to adjust for the observed variability. Further, presence of heterogeneity was also assessed by subgroup analysis. However, the level of heterogeneity was high after subgroup analysis (Figure 3). Then we further try to investigate the sources heterogeneity using metaregression model using publication year and sample size as covariates. Meta‐regression is a more complex and preferable method than subgroup analysis for investigating heterogeneity and has the potential advantage of allowing the assessment of one or more covariates simultaneously [38]. The result of metaregression analysis showed that both covariates were not statistically significant for the presence of heterogeneity (Table 2).

### 3.5. Publication Bias

**Figure 4:**
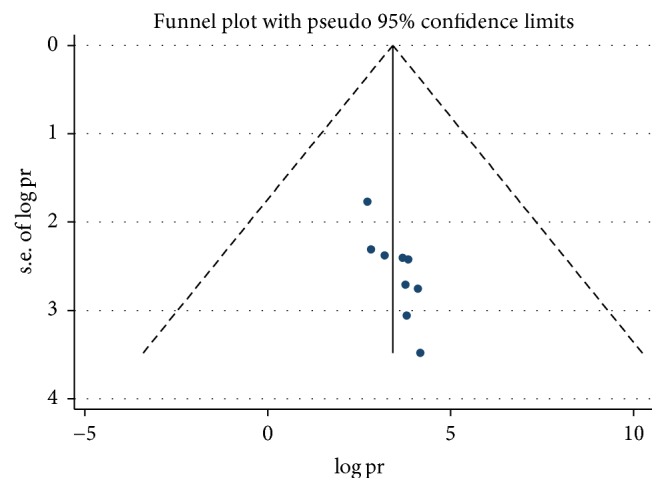
Funnel plots to test the publication bias of the 9 studies, 2018.

**Figure 5:**
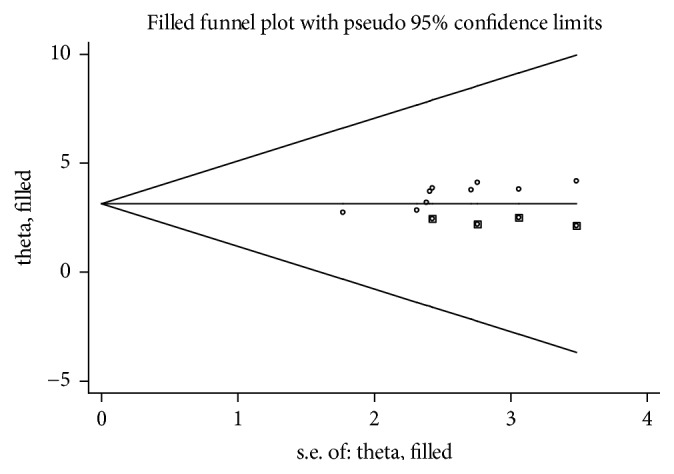
Result of trim and fill analysis for adjusting publication bias of the 9 studies, 2018.

Presence of publication bias was examined using funnel plots and tests (Egger's and begs). In this meta-analysis funnel plots and tests indicated evidence of publication bias. Each point in funnel plots represents a separate study and asymmetrical distribution is evidence of the existence of publication bias [39]. First, each study's effect size was plotted against the standard error and visual inspection of the funnel plot suggests some asymmetry, as three studies lay on the left side and six studies on the right side of the line representing the pooled prevalence (Figure 4). In addition, trim and fill analysis was also performed (Figure 5). Trim and fill analysis is a nonparametric methods for estimating the number of missing studies that might exist and it helps in reducing and adjusting publication bias in meta-analysis [40]. We also performed Egger's and Begg's tests to investigate publication bias. The result of these tests showed significant evidence of publication bias (*p* value < 0.05).

### 3.6. Sensitivity Analysis

**Figure 6:**
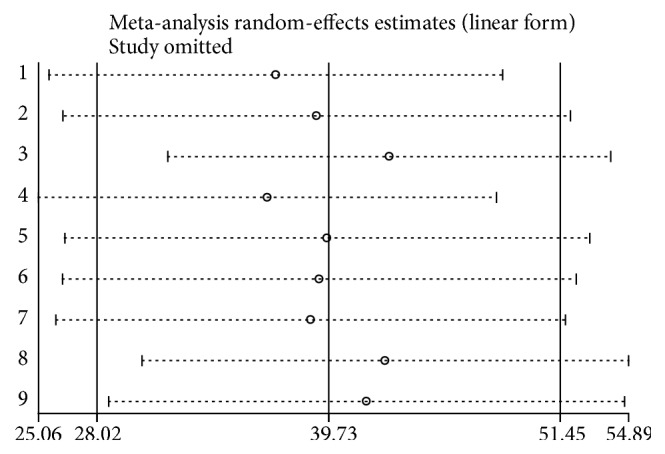
Result of Sensitivity analysis of the 9 studies, 2018.

The result of sensitivity analyses using random effects model suggested that no single study unduly influenced the overall prevalence estimate of depression among patients with diabetes (Figure 6).

## 4. Discussion

Depression is a very common highly prevalent comorbid condition in diabetic patients [41]. Comorbid depression with diabetes linked with 1.5-fold increase in risk of mortality and 50–75% increases the cost of health care service [28, 42]. There was limited data on depression as a comorbid with other chronic diseases. This meta-analysis aimed to estimate the overall pooled prevalence of depression among diabetic patients in Ethiopia.

The finding of these 9 studies involving 2944 participants showed that the overall pooled prevalence of depression among diabetic patients was 39.73% (95% CI (28.02%, 51.45%)). This finding is consistent with the studies conducted in Netherlands (31%) [43], in Bahrain (33.3) [44], in Malaysia (30.5) [45], in Palestine (40%) [46], and in Nigerian Teaching Hospital (30%) [47].

The finding of this meta-analysis showed that depression is common comorbid condition among diabetic patients. This is in line with the recent meta-analysis done in Washington [13] which identified the link between depression and diabetes mellitus and showed that the presence of diabetes doubles the odds of comorbid depression.

The pooled estimate of this meta-analysis is higher than other similar systematic reviews and meta-analyses [16, 17]. The higher prevalence of depression in Ethiopia could be due to poor quality of diabetic care service, lower level of education, measurement tool used to quantify the level of depression, and other forms of stressors. On the contrary, several studies among diabetic patients had found higher prevalence of depression than our study [48–50]. The possible reason for the lower proportion of depression might be due to strong family and social support implemented in our society. The difference might also be due to methodological issue, sample size, sociodemographic characteristics of the study participants, and others.

Although the power to detect much differences was low due to the analysis which involves small number of studies, the pooled estimate indicated that the risk of depression is very common in diabetic patients as compared with the general population. Even though there are a number of factors in each study such as age, sex, socioeconomic status, presence complications, additional comorbid condition, duration of the diseases, and the like, it was not possible to conduct factor analysis and examine the pooled odds ratios because of luck of similar and related factors across the studies.

Nevertheless, this review has provided valuable information regarding the prevalence of depression among diabetic patients, and there were some limitations that could be addressed in future systematic review and meta-analysis. One of the limitations of this study is that factor analysis was not conducted due to luck of similar factors to be analyzed across the studies as only small number of studies reviewed.

## 5. Conclusion

The systematic review and meta-analysis showed that the prevalence of comorbid depression was highly prevalent among patients with diabetes. The difference was observed in the prevalence of depression in different region of the country. The highest prevalence of depression was found in studies conducted in Addis Ababa, followed by studies conducted in Oromia region. Health education and patient education given by health care providers at all level should incorporate mental health issues among patients with diabetes mellitus. In order to improve the patient's mental status early screening with appropriate psychiatric assessments and treatment with pharmacotherapy or psychotherapy should be incorporated as a routine practice in diabetic clinic. Future research should focus on identification of the associated factors for depression among diabetic patients.

## Figures and Tables

**Table 1:** Characteristics of studies included in meta-analysis of the prevalence of depression among diabetic patients in Ethiopia, 2018.

S. number	Author/s (reference)	Publication year	Study design	Study area, region	Sample size	Response rate	Prevalence% (95% CI)
1	Erkie et al. [31]	2013	Cross-sectional	Addis Ababa	313	100%	61 (55.6, 66.4)
2	Teshager and Giru [33]	2016	Cross-sectional	Bahir Dar, Amhara region	416	100%	40.4 (35.7, 45)
3	Tilahune et al. [34]	2016	Cross-sectional	Mekele, Tigray region	264	100%	17 (12.5, 21.5)
4	Tiki [12]	2017	Cross-sectional	Ambo, Oromia region	423	100%	47 (42, 51.8)
5	Habtewold et al. [20]	2016	Cross-sectional	Addis Ababa	264	95.6%	44.7 (38.7, 50.7)
6	Birhanu et al. [18]	2016	Cross-sectional	Gonder, Amhara region	415	98%	15.4 (11.9, 18.9)
7	Tilahune et al. [35]	2016	Cross-sectional	Hawassa, SNNPR region	326	100%	24.5 (19.8, 29)
8	Dejene et al. [19]	2014	Cross-sectional	Jimma, Oromia region	335	96%	43.6 (38.3, 48.9)
9	Dessie et al. [32]	2017	Cross-sectional	Debre Markos, Amhara region	188	98%	64.9 (58, 71.7)

**Table 2:** Meta-regression analysis of factors with heterogeneity of the prevalence of depression among diabetic patients in Ethiopia, 2018.

Heterogeneity source	Coefficients	Std. err.	*p* value
Publication year	−2.886568	5.008484	0.585
Sample size	−.076825	.0818828	0.384
